# Parinaud’s syndrome in posterior stroke treated with intravenous thrombolysis: A case report

**DOI:** 10.5339/qmj.2026.41

**Published:** 2026-06-16

**Authors:** Mauricio Benetti, Fabio Gonzalez, Francisco Caiza-Zambrano, Yemina Neme-Segura, Patxi Zavala-Gottau, Pablo Bonardo

**Affiliations:** 1 Neurology Department, British Hospital of Buenos Aires, Buenos Aires, Argentina

**Keywords:** Parinaud’s syndrome, vertical gaze palsy, dorsal midbrain, posterior circulation stroke, intravenous thrombolysis, Argentina

## Abstract

**Background:**

Parinaud’s syndrome, or dorsal midbrain syndrome, is characterized by vertical gaze palsy, light-near dissociation, convergence-retraction nystagmus, and bilateral eyelid retraction. The majority of cases are caused by pineal region tumors, whereas vascular etiologies are less frequent. Early recognition of this neuro-ophthalmological syndrome can be crucial for diagnosing brainstem strokes, particularly when initial neuroimaging findings are normal.

**Case presentation:**

A 66-year-old man presented to the Emergency Department with sudden-onset vertical diplopia, dysarthria, and gait instability, consistent with an acute neurological event. Neuro-ophthalmological examination revealed the complete classical triad of Parinaud’s syndrome. Brain magnetic resonance imaging (MRI) and computed tomography angiography (CTA) showed no acute ischemic lesions or vascular abnormalities. Given the disabling oculomotor findings and the strong suspicion of posterior circulation ischemia, intravenous thrombolysis with recombinant tissue plasminogen activator (rtPA) was administered within 3.5 h of symptom onset. The patient achieved complete neurological and oculomotor recovery within 24 h, and follow-up brain MRI confirmed the absence of structural damage.

**Discussion:**

The present case illustrates Parinaud’s syndrome, a rare neuro-ophthalmological manifestation of acute posterior circulation ischemia, treated with intravenous thrombolysis. Although its global prevalence remains unknown, published series show that the syndrome is exceptionally uncommon, with vascular etiologies representing approximately 25% of cases—making stroke the second most common cause after pineal region tumors.

In contrast to pineal region tumors, which typically produce a slowly progressive clinical course, vascular etiologies are characterized by abrupt symptom onset.

**Conclusion:**

This case underscores the importance of recognizing Parinaud’s syndrome as a possible manifestation of posterior circulation ischemia, particularly when early neuroimaging findings appear normal. Its rarity and atypical presentation can make therapeutic decision-making challenging in the acute setting, highlighting the diagnostic value of a meticulous ocular examination.

## 1. INTRODUCTION

Dorsal midbrain syndrome, also known as Parinaud’s syndrome—named after the French ophthalmologist Henri Parinaud (1844–1905)—is defined as a constellation of oculomotor disturbances and pupillary dysfunction, including vertical gaze palsy, light-near dissociation, convergence-retraction nystagmus, and bilateral upper eyelid retraction (Collier’s sign), often accompanied by ocular misalignment.^1^ The most common causes of this syndrome are the pineal region tumors, followed by the cerebrovascular accidents (strokes), in which lesions usually involve the dorsal midbrain, thalamus, or pontomesencephalic junction—structures involved in the control of vertical gaze.^1–2^

Although its true population prevalence remains unknown, the largest contemporary series identified only 75 patients over 21 years, underscoring the exceptional rarity of Parinaud’s syndrome. In this cohort, neoplasms were the most frequent etiology (47%), followed by cerebrovascular events (25%).^1^ The series included patients across a wide age range—from infancy to advanced age (4 months to 86 years)—although pediatric cases were uncommon (with only 7% of patients below 10 years). Notably, a distinct age-related etiological pattern was observed: neoplasms predominantly affected younger patients (median age: 19 years), whereas cerebrovascular events were exclusively observed in adults (median age: 63 years; range: 18–83 years).

We report the case of an adult patient who presented with the sudden onset of neurological and ocular symptoms consistent with Parinaud’s syndrome secondary to an ischemic cerebrovascular event.

## 2. CASE PRESENTATION

A 66-year-old man with a history of metabolic syndrome and a previous midbrain ischemic stroke six years earlier (2019), without residual neurological deficits and not receiving chronic antithrombotic therapy related to that event at the time of presentation, presented to the Emergency Department with acute-onset vertical diplopia, dysarthria, and gait instability that had evolved over 3.5 h.

**Figure 1.**
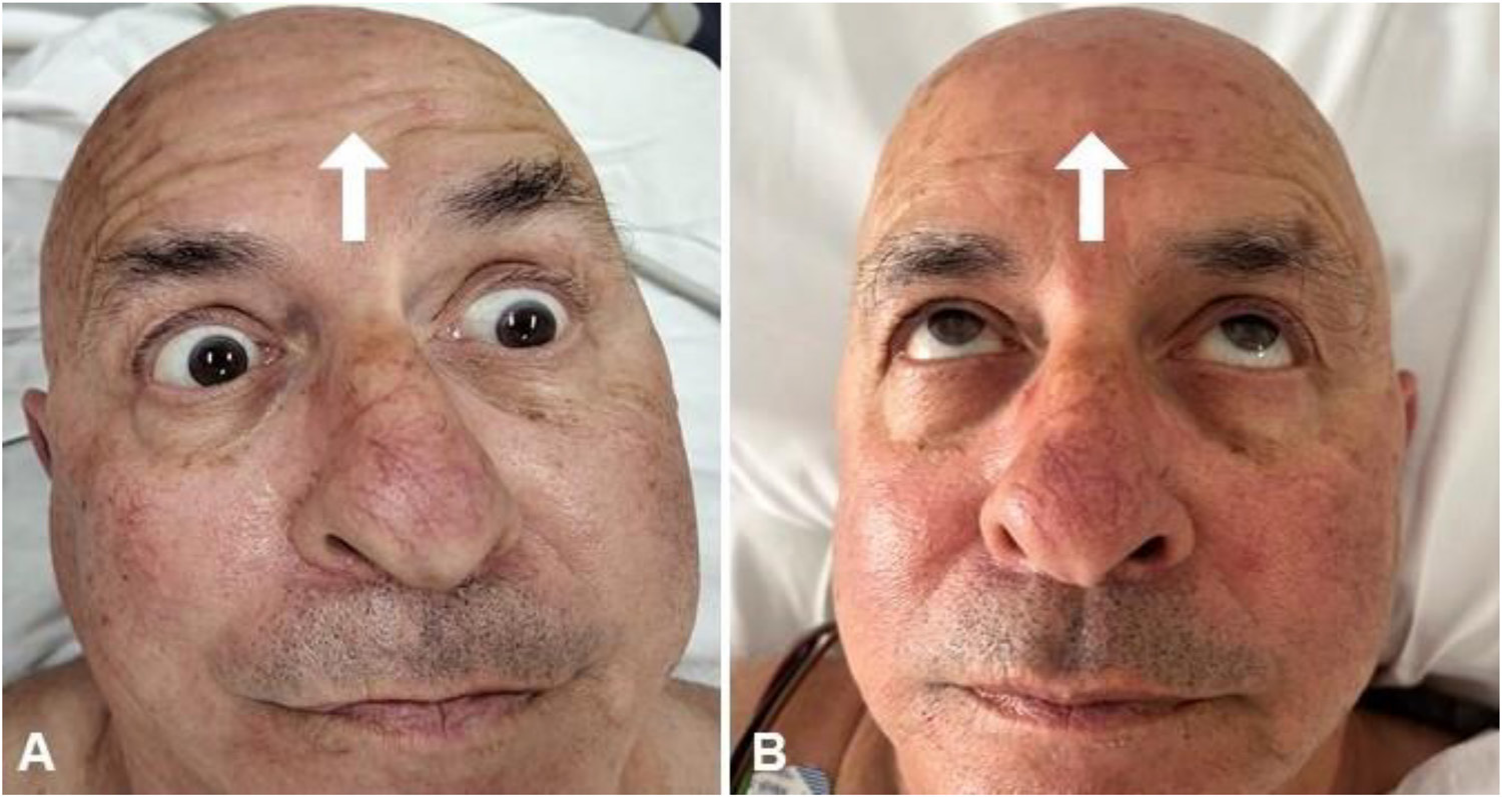
(A) Upward gaze palsy with Collier’s sign (bilateral upper eyelid retraction) on attempted supraversion prior to intravenous thrombolysis. (B) Complete resolution of vertical gaze palsy following intravenous thrombolysis, with restoration of normal upward gaze.

Neuro-ophthalmological examination revealed upward gaze palsy, absent pupillary light reflexes with preserved response to convergence (light-near dissociation), convergence-retraction nystagmus, and bilateral upper eyelid retraction (Collier’s sign), findings consistent with Parinaud’s syndrome (Figure 1A). The patient was evaluated by the emergency medicine, neurology, and ophthalmology teams. In addition, mild dysarthria and right lower-limb ataxia were noted, with an initial NIHSS (National Institutes of Health Stroke Scale) score of 3. The NIHSS is a validated tool used to quantify neurological deficit severity in acute stroke, with scores ranging from 0 (no deficit) to 42 (maximal deficit).^3^ Baseline vital signs were blood pressure 148/86 mmHg, heart rate 82 bpm, oxygen saturation 98% on room air, and temperature 36.7°C.

Brain magnetic resonance imaging (MRI) and computed tomography angiography (CTA) of the neck vessels and the circle of Willis showed no acute ischemic lesions or vascular abnormalities. Nevertheless, given the strong clinical suspicion of a posterior circulation ischemic event and the functionally disabling nature of the brainstem symptoms, intravenous thrombolysis with recombinant tissue plasminogen activator (rtPA, 0.9 mg/kg; total dose: 74 mg) was administered after exclusion of absolute and relative contraindications, in accordance with current international guidelines for the management of acute ischemic stroke.^3^

Twenty-four hours after treatment, the patient showed complete resolution of both neurological and oculomotor deficits, including recovery of vertical gaze (Figure 1B). Forty-eight hours after clinical improvement, follow-up MRI confirmed the absence of recent ischemic lesions.

Transesophageal echocardiogram revealed mild left atrial enlargement, while 24-h Holter monitoring showed no arrhythmias.

The patient was discharged 72 h after initial presentation with an NIHSS score of 0, on secondary prevention therapy with aspirin 100 mg/day and rosuvastatin 20 mg/day, prescribed indefinitely with reassessment planned during follow-up.

At one-month follow-up, he remained asymptomatic, with no recurrence of visual or neurological symptoms.

## 3. DISCUSSION

Parinaud’s syndrome usually results from a lesion of the dorsal midbrain that affects the vertical gaze centers and supranuclear pupillary pathways; however, it may also be observed in paramedian thalamic or thalamo-mesencephalic lesions that interrupt the fibers projecting toward the posterior commissure.^4,5^ From a pathophysiological standpoint, ischemic lesions in this region typically arise from partial or transient occlusion of the paramedian perforating arteries, which are terminal branches of the posterior cerebral artery or the superior basilar artery. The terminal blood supply of the dorsal midbrain—dependent on the posterior choroidal and vertebrobasilar perforating arteries—renders it particularly vulnerable to small but clinically expressive infarcts.^6^

Key structures involved in vertical gaze control include the rostral interstitial nucleus of the medial longitudinal fasciculus, the interstitial nucleus of Cajal, and the posterior commissure—located in the dorsal midbrain beneath the pineal gland—responsible for the coordination of conjugate vertical eye movements.^4^ The fibers mediating upgaze decussate through the posterior commissure, explaining why lesions in this area predominantly cause upward gaze palsy.^2,5^ Convergence-retraction nystagmus results from simultaneous co-contraction of the horizontal rectus muscles during attempted upgaze, secondary to loss of midbrain inhibitory control.^4,7^ Light-near dissociation is explained by involvement of the dorsal fibers of the pupillary light reflex projecting to the Edinger–Westphal nucleus, with sparing of the ventral fibers subserving accommodation.^5,7^ Bilateral eyelid retraction (Collier’s sign) arises from disinhibition of the levator palpebrae superioris nucleus at the level of the posterior commissure.^7^

Only about 65% of patients present with the classical triad of vertical gaze palsy, convergence-retraction nystagmus, and light-near dissociation;^8^ our patient exhibited the complete triad. Regarding etiology, a recent review of 75 cases of dorsal midbrain syndrome identified neoplasms (47%) and cerebrovascular events (25%)—mainly thalamic or midbrain in origin—as the most frequent causes, followed by non-neoplastic masses, hydrocephalus, and trauma.^1^ Our case fits within the vascular etiology, compatible with a posterior circulation ischemic event, probably secondary to small-vessel disease, with presumed dorsal midbrain localization based on clinical and neuroanatomical correlation. Although both the initial and follow-up MRI scans failed to show acute ischemic changes, the clinical presentation and topographic correlation were highly consistent with a dorsal midbrain infarct. The absence of diffusion restriction on diffusion-weighted imaging (DWI) in the initial MRI likely reflects the early timing of the scan, while the subsequent normal imaging can be explained by early recanalization and tissue reperfusion following prompt intravenous thrombolysis, preventing cytotoxic edema formation.

In posterior fossa strokes, DWI may be falsely negative, particularly in the early stages. In a prospective series of 401 patients with stroke or transient ischemic attack imaged within 24 h, up to 25% had an initially negative DWI, more frequently in lacunar and posterior circulation syndromes.^9^ Consistently, a subsequent meta-analysis including only confirmed ischemic strokes estimated a pooled prevalence of DWI-negative stroke of 6.8%, with a five-fold higher likelihood in posterior circulation events.^10^

Early recognition of the oculomotor signs of Parinaud’s syndrome is essential for the rapid suspicion of ischemic lesions in the midbrain or adjacent regions, even when initial neuroimaging findings are normal. In our case, the detection of a functionally disabling oculomotor presentation—characterized by upward gaze palsy and diplopia—despite a low NIHSS score, supported the decision to perform intravenous thrombolysis in the absence of contraindications, leading to complete symptom resolution within 24 h.^3^

To our knowledge, this is the first reported case of Parinaud’s syndrome associated with an acute ischemic stroke successfully treated with intravenous thrombolysis, resulting in complete clinical recovery. Only one previous case has described dorsal midbrain syndrome treated with intraventricular thrombolysis directed at a clot compressing the dorsal midbrain within the cerebral aqueduct.^11^ In that report, Parinaud’s syndrome resulted from direct compression of the dorsal midbrain by an intraventricular clot. The patient received intraventricular fibrinolysis with rtPA through an external ventricular drain for three consecutive days (total dose 5 mg), and the syndrome resolved completely within 48 h after the final dose. This differs from our patient, whose presentation was consistent with an ischemic mechanism and who achieved complete clinical recovery within 24 h following systemic intravenous thrombolysis rather than intraventricular therapy.

This case illustrates how a classical neuro-ophthalmological syndrome can represent the initial manifestation of an acute cerebrovascular event, underscoring the diagnostic value of ocular findings in the topographic localization of neurological damage.

## 4. CONCLUSION

We reported a case of Parinaud’s syndrome as the initial manifestation of a posterior circulation ischemic stroke, treated with intravenous thrombolysis and resulting in complete neurological recovery. Although stroke is a recognized cause of Parinaud’s syndrome, its presentation predominantly as an isolated vertical gaze abnormality remains uncommon.

This case highlights the importance of early clinical recognition and interdisciplinary collaboration among emergency physicians, neurologists, and ophthalmologists to ensure timely diagnosis and treatment, thereby optimizing the neurological and functional outcomes.

## DATA AVAILABILITY

Data are available from the corresponding author upon reasonable request.

## ETHICAL APPROVAL

This article describes a single clinical case. All procedures complied with national regulations and institutional policies, and were conducted in accordance with the principles of the Declaration of Helsinki. Institutional review board approval was not required for this type of publication. Written informed consent was obtained from the patient for publication of this case report and the accompanying clinical photograph showing his face.

## AUTHOR CONTRIBUTIONS

MB contributed to study conception and design, patient evaluation, data collection, drafting of the manuscript, and critical revision of the manuscript for important intellectual content. FG, FC-Z, YN-S., PZ-G., and PB contributed to data interpretation, critical revision of the manuscript for important intellectual content, and approved the final version of the manuscript.

## FUNDING

The author(s) received no financial support for the research, authorship, and/or publication of this article.

## ACKNOWLEDGMENTS

The author(s) have no acknowledgements to declare.

## DISCLOSURE OF AI USE

The authors declare that no generative AI tools were used in the preparation of this manuscript.

## COMPETING INTERESTS

The author(s) declare that there is no conflict of interest.

## References

[1] Yousif JE, Liao E, Trobe JD (2021). Dorsal midbrain syndrome: Clinical and imaging features in 75 cases. J Neuroophthalmol.

[2] Gupta A, Singh AS (2022). Un caso raro de síndrome de Parinaud con astasia. Delta J Ophthalmol.

[3] Powers WJ, Rabinstein AA, Ackerson T, Adeoye OM, Bambakidis NC, Becker K (2019). Guidelines for the early management of patients with acute ischemic stroke: 2019 update to the 2018 guidelines for the early management of acute ischemic stroke: A guideline for healthcare professionals from the American Heart Association/American Stroke Association. Stroke.

[4] Serino J, Martins J, Páris L, Duarte A, Ribeiro I (2015). Parinaud’s syndrome due to an unilateral vascular ischemic lesion. Int Ophthalmol.

[5] Alrawashdeh HM, Al-Habahbeh O, Al-Salem M (2020;). Reversible Parinaud’s syndrome during the first 24 hours following a transient ischemic attack: A case report and review of literature. Neurol Asia.

[6] Gowda SN, Munakomi S, De Jesus O (2025). Brainstem stroke. In: StatPearls [online].

[7] Prakash E, Kuht HJ, Harieaswar S, Thomas MG (2021). Dorsal midbrain (Parinaud) syndrome. Pract Neurol.

[8] Magdum R, Aher PS (2024). Parinaud syndrome in a patient with microangiopathic lesions in the bilateral gangliocapsular region and left thalamus. Cureus.

[9] Sylaja PN, Coutts SB, Krol A, Hill MD, Demchuk AM, VISION Study Group (2008). When to expect negative diffusion-weighted images in stroke and transient ischemic attack. Stroke.

[10] Edlow BL, Hurwitz S, Edlow JA (2017). Diagnosis of DWI-negative acute ischemic stroke: A meta-analysis. Neurology.

[11] Laforce R, Khuong H, Gariépy JL, Milot G, Savard M (2009). Reversible Parinaud syndrome following intraventricular thrombolysis. Can J Neurol Sci.

